# Hybrid Laser Deposition of Composite WC-Ni Layers with Forced Local Cryogenic Cooling

**DOI:** 10.3390/ma14154312

**Published:** 2021-08-02

**Authors:** Aleksander Lisiecki, Dawid Ślizak

**Affiliations:** 1 Department of Welding Engineering, Faculty of Mechanical Engineering, Silesian University of Technology, Konarskiego 18A Str., 44-100 Gliwice, Poland; 2 Additive Manufacturing Laboratory, PROGRESJA S.A., Żelazna 9 Str., 40-851 Katowice, Poland; dslizak@progresja.co

**Keywords:** laser cladding, laser deposition, hybrid process, cryogenic conditions, composite coatings, WC-Ni coatings, fiber laser

## Abstract

The purpose of this study was to demonstrate the effect of forced and localized cooling by nitrogen vapours stream under cryogenic conditions during laser deposition of WC-Ni powder on the geometry, microstructure of clad layers and dry sliding wear resistance of the coatings. For this purpose, comparative tests were performed by conventional laser cladding at free cooling conditions in ambient air and by the developed novel process of laser deposition with additional localized cooling of the solidifying deposit by nitrogen vapours stream. Due to presence of gaseous nitrogen in the region of the melt pool and solidifying deposit, the process was considered as combining laser cladding and laser gas nitriding (performed simultaneously), thus the hybrid process. The influence of the heat input and cooling conditions on the geometrical features, dilution rate, share of carbides relative to the matrix, and the fraction share of carbides, as well as hardness profiles on cross sections of single stringer beads was analysed and presented. The XRD, EDS analysis and the sieve test of the experimental powder were used to characterize the composite WC-Ni type powder. The OM, SEM, EDS and XRD test methods were used to study the microstructure, chemical and phase composition of clad layers. Additionally, ball-on-disc tests were performed to determine the wear resistance of representative coatings under dry sliding conditions. The results indicate that the novel demonstrated technique of localized forced cooling of the solidifying deposit has advantageous effect, because it provides approximately 20% lower penetration depth and dilution, decreases tendency for tungsten carbides decomposition, provides more uniform distribution and higher share of massive eutectic W_2_C-WC carbides across the coating. While the conventionally laser cladded layers show tendency for decomposition of carbide particles and resolidifying dendritic complex carbides mainly M_2_C, M_3_C and M_7_C_3_ containing iron, nickel, and tungsten, and with Ni/Ni_3_B matrix. The quantitative relationship between heat input, cooling conditions and the carbides grain size distribution as well as carbides share in relation to the matrix was determined.

## 1. Introduction

Laser cladding offers some advantages over the other methods of cladding or coatings [1,2,3,4,5,6,7,8,9,10]. The most significant advantages are high power density, localized and precise heating, high scanning speed, thus low heat input, low penetration depth and low dilution. Laser cladding is often used to produce wear-resistant layers based on metallic and composite materials. One group of composite materials are metal matrix composites (MMC) based on nickel matrix reinforced with tungsten carbides. Such composites are characterized by good wear resistance, corrosion resistance and also satisfactory dynamic load resistance. The high abrasive wear resistance is provided by hard particles of tungsten carbides distributed in the ductile nickel matrix. In turn, the ductile matrix provides the ability to withstand high loads, especially compressive stresses. Moreover, such composites show satisfactory cohesion between hard WC phases and the matrix of Ni solution, due to good wettability of WC particles by the Ni-based alloys. The process of laser cladding is considered as advantageous for manufacturing the composite clad layers due to low heat input, thus limited reaction between the ceramic phases and the metallic matrix [11,12,13,14]. However, even in the case of laser cladding, especially in the output power range of several kilowatts, the heat input may be too high producing unfavourable microstructure due to partial and even complete melting of WC particles. Moreover, under specific conditions the WC particles tend to fall off in the melt pool, causes uneven distribution of the carbides of various size across the surface layer. Zhang et al. indicate that the accumulation of WC particles at the bottom of the clad coating is detrimental for the properties of the coating [12]. They also point that it is the biggest obstacle to engineering applications of such composite coatings. Various methods for providing the uniform distribution of ceramic particles in the composite coatings, such mechanical vibration, ultrasonic, magnetic fields were studied and described in the literature [13]. Li et al. demonstrated an original technique of laser cladding of WC-Ni composite coatings assisted by micro-vibrations generated by a vibration exciter system made of magneto strictive materials [14,15]. They pointed the beneficial effect of the micro-vibrations on microstructure, including uniform distribution of tungsten carbides [15]. In turn Huang et al. demonstrated a cladding technique involving a pulsed Nd:YAG laser for reducing the heat input [16]. They successfully produced dense and crack-free WC-Ni composite clad layers on H13 substrate with the thickness up to 1.0 mm.

Another technique that allows to reduce the effect of the heat on the material and to control the solidification rate is a forced cooling. An original technique of micro-jet cooling by compressed gas streams of the deposit during arc cladding was elaborated and demonstrated by Węgrzyn et al. [17]. They proved the beneficial effect of the forced cooling of the deposit at solidification stage resulted in refinement of microstructure and enhanced wear resistance.

Liquid nitrogen bath providing cryogenic conditions of cooling was also tested by some researchers during laser surface melting or heat treatment, mainly for nonferrous alloys [18,19,20,21,22,23]. Such technique of liquid nitrogen bath cooling of the substrate was adopted and developed by the authors for laser powder deposition of metallic and composite coatings, as demonstrated in several previous publications [1,24,25,26,27,28,29,30,31]. It is worth to note, that complexity and difficulty of the laser powder deposition under such cryogenic conditions with coaxial powder delivery into the melt pool is significantly higher than the laser surface melting of the substrate supercooled by liquid nitrogen bath. Moreover, the results obtained so far indicate that due to intensive evaporating of liquid nitrogen, the gaseous nitrogen present in the region of powder deposition and melt pool provides an active atmosphere typical for laser gas nitriding (LGN) or laser alloying (LA). Therefore, the proposed new method of laser coating is considered as a hybrid process combining laser powder deposition and laser gas nitriding, additionally conducted under cryogenic conditions.

The preliminary tests of laser cladding of composite WC-Ni coatings, conducted by the authors, have shown that the forced cooling of the substrate by liquid nitrogen bath can provide some beneficial effects. The most significant are limited penetration depth, limited dilution, limited tendency for tungsten carbide melting, uniform and dense distribution of carbides, favourable hardness distribution and high values of hardness. However, some disadvantages and limitations of the liquid nitrogen bath (volume cooling) applied for laser powder deposition were identified. Due to high extent of supercooling the substrate and intensive dissipation of the heat during laser heating, the tendency for incomplete or lack of penetration of the clad was observed, especially at the lowest heat input range. Therefore, another technique of forced cooling by means of liquid nitrogen based on stream local cooling of the deposition region, that allows controlled and less intensive cooling of the substrate was developed and investigated by the authors.

According to the authors’ knowledge and experience, the presented results are fully original and unique, and the results of similar studies have not been available nor published so far.

## 2. Materials and Methods

### 2.1. Materials

The non-alloy structural steel S235JR (according to EN 10025-2) was chosen as the substrate for technological tests. This grade of steel with a low content of alloying elements, good plasticity and excellent weldability was chosen to minimize the influence of the substrate material on the cladded layer, especially minimize the internal stresses and tendency for cracking of clad layers, Table 1. Specimens for laser cladding tests were cut from a steel plate 5.0 mm thick into coupons with dimensions 100 × 100 mm^2^.

**Figure 1:**
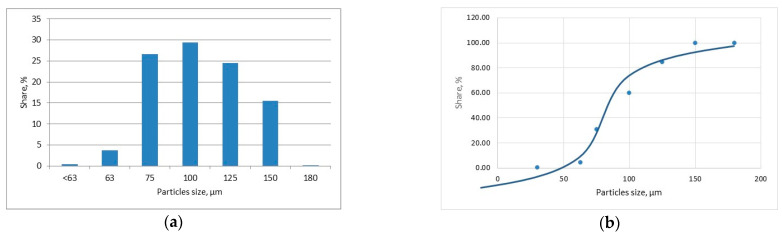
The mass fraction share (**a**) and cumulative particle size distribution curve (**b**) determined by sieve test (PN-EN 24497/ISO 4497) for the experimental composite WC-Ni powder, Table 2.

**Figure 2:**
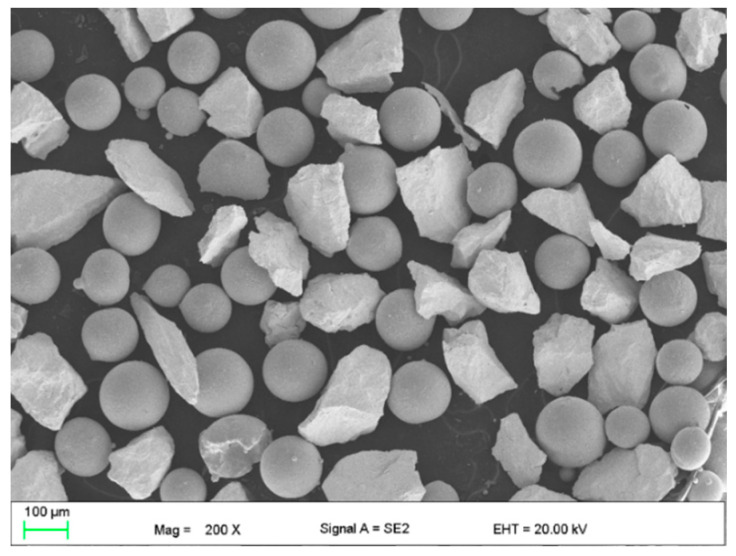
Morphology of the experimental composite WC-Ni powder; mixture of irregular WC particles and spherical metallic Ni-based particles, Table 2.

**Figure 3:**
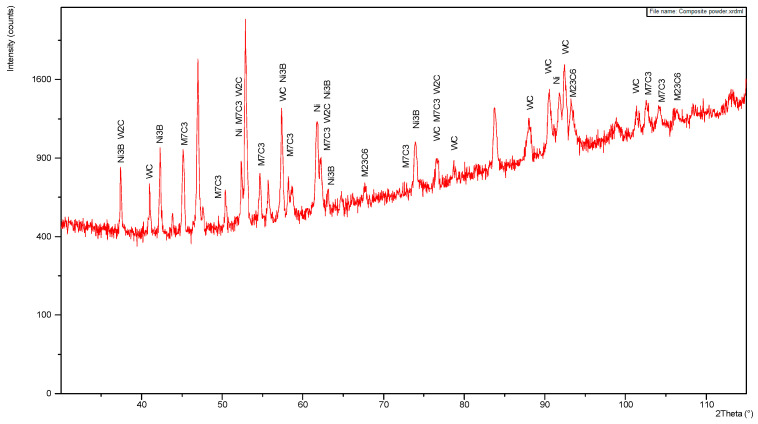
XRD pattern of the experimental WC-Ni composite powder, Table 2, Figure 1 and Figure 2.

In turn, the powder for cladding was experimentally composed in such a way as to ensure high abrasion resistance, and corrosion resistance of deposit even at elevated temperatures. Therefore, tungsten carbides, as the reinforcing phase, were mixed with nickel-based powder providing the ductile metal matrix characterized by high resistance for corrosion and high temperature. The nickel-based metal powder was gas atomized in argon atmosphere and the spherical particles size range was 63 ÷ 160 µm. While the tungsten carbide irregular shaped (crushed) particles size range was 100 ÷ 160 µm. The sieve test of the mixed experimental powder was performed for statistical analysis according to the standard PN-EN 24497/ISO 4497. Sieve shaker LPzE-2e (Multiserw, Brzeźnica, Poland) and the moisture analyser with the laboratory scales BTS110 (AXIS, Gdańsk, Poland) were applied in the sieve test. The sieve test was repeated three times for different batches of powder weighing approx. 100 g each, for statistical reasons. The mass fraction share and cumulative particle size distribution curve determined for the experimentally composed powder are presented in Figure 1. The morphology, chemical and phase composition of the experimental powder are presented in Figure 2 and Figure 3.

### 2.2. Laser Deposition Tests

The tests of laser deposition were performed by means of a prototype robotized stand. The stand was equipped with a six-axis robot Panasonic GII TL-190 (Industrial Solutions Company, Panasonic Corporation, Osaka, Japan) with maximum load capacity 6.0 kg, high power fiber laser (HPFL) IPG YLS-3000-CT-Y15 (IPG Photonics, Oxford, MS, USA) with maximum output power 3.0 kW, emitting at a wavelength 1.07 µm, and characterized by a single mode energy distribution across the spot (Gaussian intensity distribution), custom made powder delivery system, and a specially designed system for localized delivery of liquid nitrogen or nitrogen vapours stream. The laser beam was transmitted from the laser generator to the focussing head IPG FLW by 20 m long HLC-8 fiber with a core diameter 200 µm. The focal length of the applied FLW head was 200 mm, while the collimator focal length was 100 mm. The beam parameter product (BPP) of the laser beam determined for the applied optics configuration was 5.8 mm⋅mrad. The BPP was measured by Primes Focus Monitor FM-120 (PRIMES GmbH, Pfungstadt, Germany).

**Figure 4:**
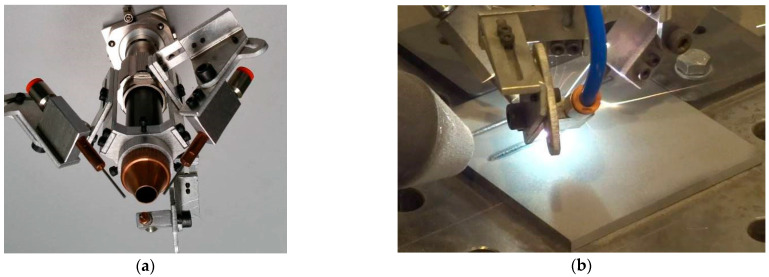
(**a**) A view of the mount of three coaxial nozzles for powder delivery and injection into the melt pool on the laser focusing head; and (**b**) a view of the hybrid laser deposition of composite WC-Ni powder at cryogenic conditions by the localized cooling with the nitrogen vapours stream and the head with three coaxial nozzles.

**Figure 5:**
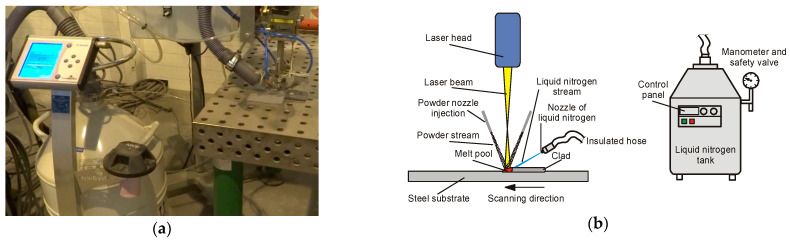
(**a**) A view of the experimental setup for laser powder deposition of composite layers with forced local cryogenic cooling by the nitrogen vapours stream (from left: cryogenic dewar with automatic control unit, elastic hose for nitrogen delivers, nitrogen stream nozzle and laser head with coaxial nozzles for powder injection); (**b**) a scheme of the laser powder deposition with forced cooling of the substrate by localized nitrogen vapours stream.

The stream localized cooling system was designed based on the experiences of previous preliminary study of laser cladding the steel substrate immersed partially in liquid nitrogen bath, to overcome the encountered limitations of such technique of volume cryogenic cooling [1]. During the previous preliminary tests, a wide range of processing parameters was investigated, at different scanning speed and output laser power. In this study the parameters were narrowed and optimized in order to provide the width of a single bead approx. 2.0 ÷ 3.0 mm, and proper fusion to the steel substrate, Table 3, Figure 4 and Figure 5.

**Figure 6:**
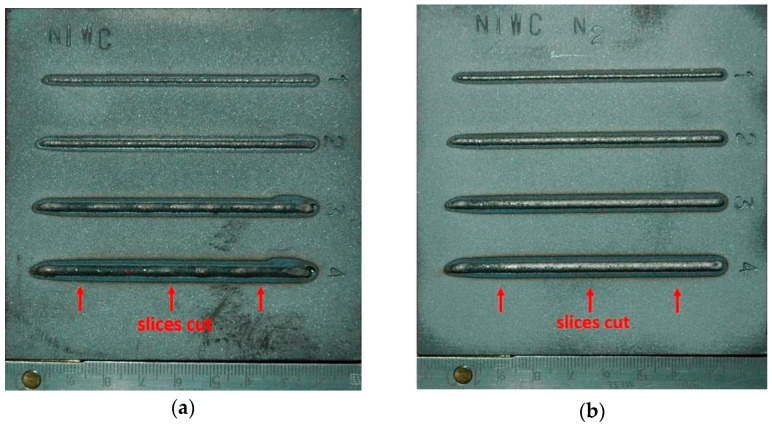
A view of stringer beads produced by laser deposition of experimental composite WC-Ni powder at free cooling conditions (conventional laser cladding); (**a**) and forced localized cooling by nitrogen vapours stream under cryogenic conditions (hybrid laser deposition process); (**b**), Table 3.

Prior to the laser cladding tests, the specimens were sandblasted (surface roughness Ra 25 ÷ 60 μm) and degreased with acetone to remove surface contamination and provide stable and repeatable surface conditions for absorption of laser radiation. Surface roughness was determined by a portable surface roughness tester SJ-210 Surftest (Mitutoyo Corporation, Kanagawa, Japan). The test cladded layers were produced as single stringer beads by laser deposition of the experimentally composed powder at different heat input and different conditions of cooling. The length of the individual bead was 80 mm, while the shift between beads was 20 mm, Figure 6. One set of test clad layers was produced during conventional laser cladding with natural cooling (so called free cooling) of the specimen in the ambient air. The second set of test clad layers was produced at the same processing parameters but additionally with the application of forced cooling by localized stream of liquid nitrogen. The temperature of the liquid nitrogen in the container was below −196 °C, while the temperature of the nitrogen vapours stream measured at the tip of the nozzle by a thermocouple was approx. −160 ÷ 164 °C. In such a way deep cryogenic treatment (DCT) was provided in the localized area of forced cooling, Table 3, Figure 4b.

The scanning speed was kept constant at 500 mm/min, while the laser output power was varied from 500 W, 1000 W, 1500 W, and 2000 W. The feeding rate of the composite WC-Ni powder was maintained constant at 8.5 g/min. The powder was fed into the melt pool by three coaxial nozzles with a diameter of 0.8 mm each. The nozzles were attached to head body providing the ability to change the angle of inclination and vertical shift, as can be seen in Figure 4a. The individual streams from the nozzles were focused on the melt pool surface in the region of laser beam interaction, Figure 4. The powder feeding rate was controlled by a rotary disc feeder PFU4 (Durum, Willich, Germany), with the chamber filled in by argon at the pressure 1.5 bar, used also as the carrier gas. The flow rate of the carrier argon gas was kept at 8.0 L/min.

The laser beam was set perpendicularly to the surface of the substrate, and it was transmitted through a 10.0 mm diameter cylindrical nozzle in argon atmosphere, at the flow rate 20.0 L/min. The argon flow was used mainly for protection the optics inside the laser head against spatter. While the task of shielding the melt pool was negligible due to long distance (160 mm) between the nozzle and the substrate surface, as a result of defocusing the laser beam spot, Figure 4b. The laser beam spot diameter was 300 µm at the applied configurations of optics. In order to provide wider area of laser beam interaction on the substrate material, the beam was defocused by lifting the laser head and thus focal plane over the top surface of the substrate. In this way, as a result of lifting the focal plane to the distance of 160 mm from the substrate, the diameter of the laser beam was approximately 3.0 mm on the top surface, Figure 4b and Figure 6.

Nitrogen vapours from a pressurized container (cryogenic dewar) were transported via a flexible hose into the jet nozzle. The nozzle tip was designed to be replaceable. Thanks to that different diameter tip nozzles could be applied. In this study a nozzle tip with a diameter of 5.0 mm was used, providing localized cooling of the clad area just behind the melt pool. The jet nozzle was fixed on the laser processing head, and the distance between the nozzle tip and the target surface was maintained constant at 40.0 mm, Figure 4b and Figure 5a. The outflow rate of the nitrogen vapours stream from the nozzle depended on the pressure inside the container, which was maintained constant by an automatic pressure control unit KRIOSAN (KrioSystem, Wrocław, Poland), Figure 5. The average consumption of liquid nitrogen was ranged in 80 ÷ 100 g/min.

### 2.3. Macro and Microstructure Examinations

The specimens with test clad layers produced both by conventional laser cladding at free cooling conditions in ambient air, and the clad layers produced at forced cooling under cryogenic conditions were examined by visual inspection first. All the clad layers fulfilled the main criterion of complete penetration of the substrate. Therefore, all the test layers were cut perpendicularly and samples for metallographic study were prepared. Three sliced sections were taken from each test clad layer. One was taken from the middle region, and next two at a distance 15 mm from the beginning and the end of the clad layer, Figure 6. The sliced sections were first mounted in thermosetting phenol resin with graphite filler Electro-WEM (Metalogis, Warsaw, Poland), and next the samples were wet grinded by water papers with grit 120 to 2500 using an automatic grinding/polishing machine Struers Labopol-2 (Struers, Rodovre, Denmark). Next the cross-sections were polished with 1 µm diamond suspension Metkon Diapat-M (Metkon Instruments Inc., Bursa, Turkey). After polishing, the cross-sections were etched by the HNO_3_ + 3HCL reagent to disclose the microstructure.

Observations of macrostructure at low magnifications (up to 25×) were carried out by means of a stereoscopic microscope OLYMPUS SZX9 (Olumpus Corporation, Tokyo, Japan), while the microstructure observations were done by the inverted metallographic microscope NIKON Eclipse MA100 (Nikon Corporation, Tokyo, Japan). The microstructure was additionally examined by scanning electron microscopy SEM (Carl Zeiss, Oberkochen, Germany), equipped with the Energy Dispersive Spectrometer EDS (Oxford Instruments, Abingdon, GB, USA) and Phenom Pro-X SEM equipped with EDS and BSD detectors (Thermo Fisher Scientific, Eindhoven, The Netherlands). The phase composition was determined by X-Ray diffraction (Panalitycal, Almelo, The Netherlands) with CuKα source of radiation, with the scanning range of the diffraction angle 2θ from 0 to 140°.

### 2.4. Hardness Measerements

The hardness was measured on the cross-section of the test clad layers after microstructural studies by Vickers test, at the load 5 N and the dwell time 10 s. The hardness tester WILSON WOLPERT 401 MVD (Wolpert Wilson Instruments, Aachen, Germany) was applied in the study. Measurements were taken along the vertical axis of symmetry, starting from the under surface region of the clad layer (face of the clad). The first measuring point was 0.15 mm under the top surface, while the distance between the subsequent points was constant at 0.2 mm. In such a way, the hardness distribution from the under surface region, through the clad, fusion zone, heat affected zone, till to the base metal was determined.

### 2.5. Tribological Test

The tribological tests of coatings produced by laser deposition of experimental composite powder at free cooling and under cryogenic conditions were conducted by a ball-on-disc tribometer T-01M under room temperature of 22 °C, according to the ASTM G99 standard. The relative humidity was about 45% ± 5%. The specimens were prepared in a form of discs with diameter of 45.0 mm balls made of bearing steel (EN 100Cr6, AISI 5210) with a diameter of 10.0 mm were used as the counter face material. The normal load was set as 20 N. The number of revolutions was 1500, while radius of the track was 15 mm. Therefore, the sliding distance was 141.3 m, while the sliding speed was 0.157 m/s. The tangential force of friction and displacement value were continuously measured and recorded during tests using a data acquisition system with PC computer. While the coefficient of friction µ was calculated by dividing the value of tangential force of friction by the value of normal load used:

µ = T/F_n_, (1)

where:

F_n_—normal load (30 N), T—tangential force of friction.

## 3. Results and Discussion

### 3.1. Macrostructure and Single Bead Geometry

**Figure 7:**
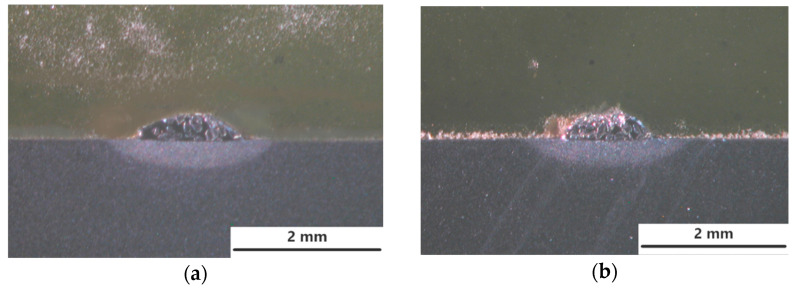

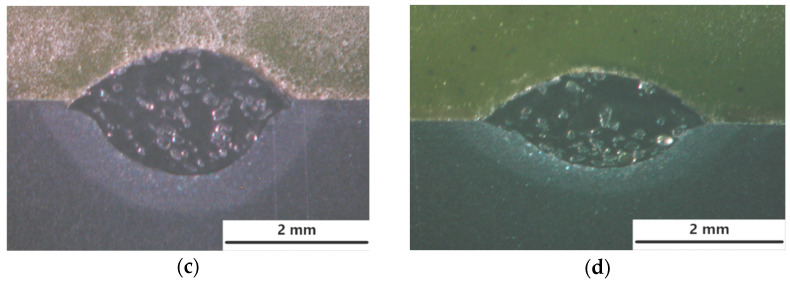
Macrostructure and the single bead geometry of the clad layers produced by laser cladding of experimental composite WC-Ni powder at constant scanning speed 500 mm/min, Table 3: (**a**) output power 500 W at free cooling; (**b**) output power 500 W at forced localized cooling; (**c**) output power 2000 W at free cooling; (**d**) 2000 W at forced localized cooling.

At the first stage of the study the cross-sections of the clad layers produced as single stringer beads by conventional laser cladding at free cooling conditions were compared with the single stringer beads produced by the novel technique considered as a hybrid laser deposition under cryogenic conditions, Figure 7 Since the clad layers were produced at constant scanning speed, the influence of energy input on the bead geometry can be also considered. The energy input is defined as follows:

Ev = P/v (J/mm),(2)

where P is the output power of laser beam (W), and v is the scanning speed (mm/s).

It is worth to note, when the heat transfer efficiency into the material is known and considered in the calculation, this parameter is called heat input. In laser processing the heat transfer efficiency is related with the absorption of laser energy, and under certain conditions it can take the value 1. Therefore, for simplicity the term “heat input” will be used in the remainder of the manuscript text. As seen in Figure 7, clear interface lines between the clads and the substrate can be distinguished, as well as the heat affected zone regions. A rough comparison of the geometry of clad layers produced at the same heat input but different cooling conditions, indicates that the forced localized cooling by nitrogen vapours stream leads to a clear decrease of penetration depth and thus dilution.

The value of dilution “*D*” was calculated by the following formula:

(3) $$D=\frac{A_{FZ}}{A_{FZ}+A_{CL}}\cdot 100\%,$$

where: *A_FZ_* is the cross-section area of the fusion zone, and the *A_CL_* is the cross-section area of the clad layer (Figure 7).

**Figure 8:**
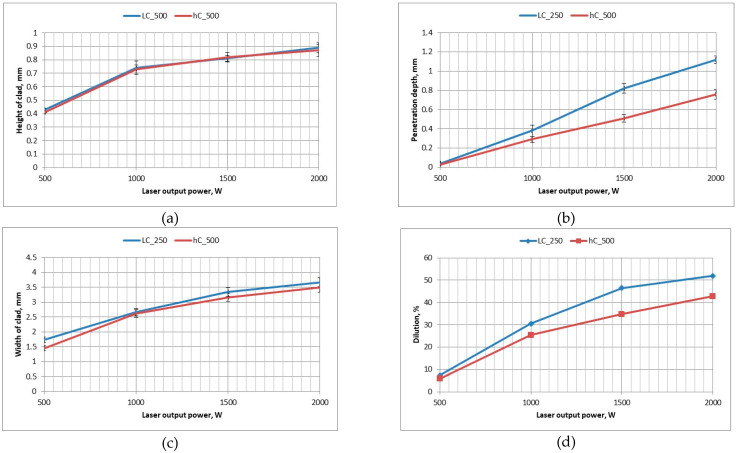
Influence of the process parameters and cooling conditions on the geometry and dimensions of the stringer beads produced by laser deposition of WC-Ni powder (Table 3): (**a**) height of the clad (reinforcement); (**b**) penetration depth into the substrate; (**c**) width of the single clad layer; and (**d**) calculated rate of dilution.

The increase in laser output power, and therefore increase in heat input, leads to an increase in the curvature of the interface line, penetration depth, height and width of the clad, Figure 7 and Figure 8.

Based on the visual inspection and analysis of cross-sections morphology, no cracks neither tendency for cracking was observed both in the clad layers produced at free cooling and under cryogenic conditions. It is related with the relative high ductility of the nickel base matrix. However, single pores were found on cross-sections of the clads produced at free cooling in the range of heat input 120 ÷ 240 J/mm. The single pore with the highest diameter of 180 µm was found on the cross-section of the clad LC3. In turn, the tendency for porosity was found to be less in the case of clads produced under cryogenic conditions. Just a single pore was found just on the cross-section of the clad produced at the highest heat input of 240 J/mm but its diameter was lower, approx. 140 µm. Thorough analysis of the geometrical parameters on cross-sections of the clads showed that the localized forced cooling of the cladding region by a stream of nitrogen vapours has neglectable influence on the height of the clads. As can be seen in Figure 8a, the height of the clads produced under different conditions of cooling (free cooling and localized forced cryogenic cooling) is comparable. In turn, the average width of the clads produced under cryogenic conditions is approximately 5 ÷ 15% lower if compared to the clads produced at free cooling (conventional laser cladding), Figure 8c. A greater difference can be noticed in the case of penetration depth and dilution, Figure 8b,d. The average penetration depth of the clads produced at the same heat input but localized forced cooling (cryogenic conditions) is 24% to 38% lower than in the case of clads produced at free cooling conditions. While the average dilution of the clads produced at localized forced cooling is 20 ÷ 25% lower if compared to the clad produced by conventional laser cladding at free cooling.

The obtained results indicate that the localized cooling of the substrate in the region of cladding can effectively reduce the penetration depth by dissipating heat. However, the applied technique of localized cooling does not significantly reduce the height and width of the clads, thus also the area of the clads. Simultaneously, the applied technique of localized cooling has a beneficial effect on reducing the penetration depth and dilution, as can be seen in Figure 8b,d. This phenomenon is caused by the localized cooling just a narrow region along the axis of the stringer bead which leads to reducing the maximum temperature in the middle region of laser beam interaction and reduces the temperature gradient.

### 3.2. Microstructure

**Figure 9:**
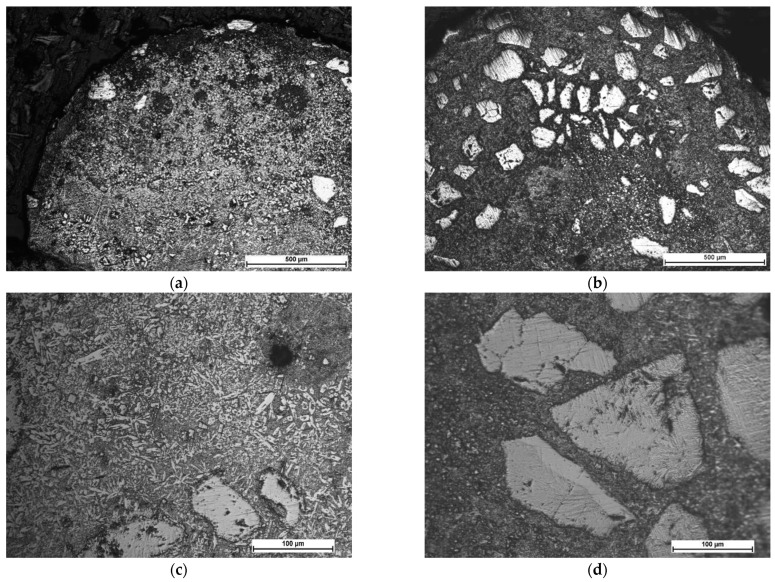
Microstructure of the comparative stringer beads LC4 and HC4 produced at the same maximum heat input of 240 J/mm (scanning speed 500 mm/min, laser output power 2000 W) but different cooling conditions (Table 3): (**a**,**c**) free cooling in ambient air—a view of the entire clad layer and the middle region of the clad respectively; and (**b**,**d**) forced localized cooling by nitrogen vapours stream under cryogenic conditions (“hybrid laser deposition”)—a view of the entire clad layer and the middle region of the clad respectively.

**Figure 10:**
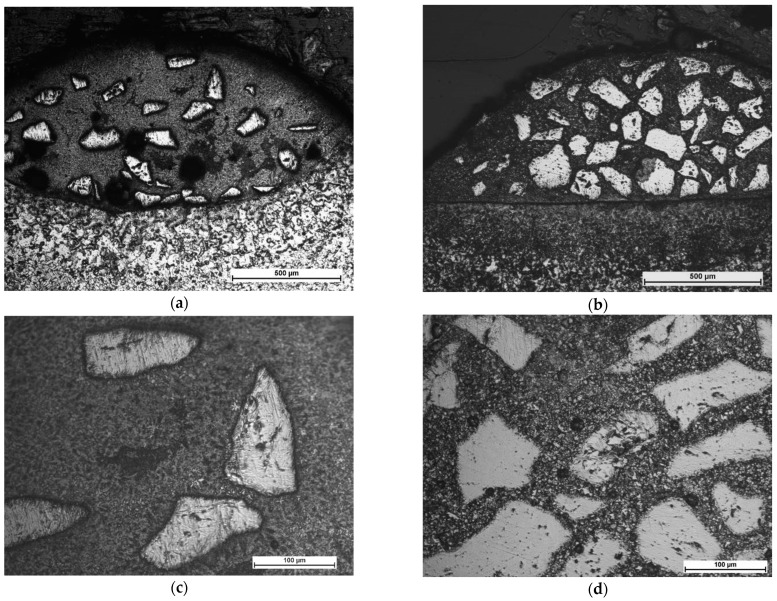
Microstructure of the comparative stringer beads LC1 and HC1 produced at the same minimum heat input of 60 J/mm (scanning speed 500 mm/min, laser output power 500 W) but different cooling conditions (Table 3): (**a**,**c**) free cooling in ambient air—a view of the entire clad layer and the middle region of the clad respectively; and (**b**,**d**) forced localized cooling by nitrogen vapours stream under cryogenic conditions (“hybrid laser deposition”)—a view of the entire clad layer and the middle region of the clad respectively.

The comparative microstructures of the clad layers produced at maximum heat input of 240 J/mm (output power 2000 W, scanning speed 500 mm/min) and different cooling conditions (LC4 and HC4) are presented on optical micrographs in Figure 9, while the microstructures of the clad layers produced at minimum heat input of 60 J/mm and different cooling conditions (LC1 and HC1) are presented on optical micrographs in Figure 10. Comparing the Figure 9a,b, it can be seen, that the number and size of the carbides are different on the cross sections of the clads produced at the same heat input but different cooling conditions. Only single massive carbides can be found on the cross section of the clad layer produced at maximum heat input and free cooling conditions. In the case of the clad layer produced at the same heat input but at forced cooling by the stream of nitrogen vapours, the number of carbides is clearly higher. Closer view of the microstructure of the clad produced at maximum heat input and free cooling conditions (LC4) revealed that the regions between massive carbides are rich mainly in needle-like precipitations with a length up to approx. 40 µm, Figure 9c. For comparison, the microstructure in the regions between massive carbides of the clad produced at the maximum heat input but at localized forced cooling is different, Figure 9c. As can be seen, the number and size of precipitation is much smaller. The longest needle-like precipitations have a length up to approx. 15 µm, Figure 9d. The reason of this phenomenon is different cooling thus different thermal conditions during deposition and solidifying of the two comparative clad layers.

The clad layer LC4 was produced by conventional laser cladding at free cooling in ambient and at relatively high heat input 240 J/mm, Table 3. Under such conditions, the massive carbides exposed to high temperature for a relatively long time, decompose and dissolve in the melt pool. It is worth noting that in addition to being exposed to the thermal influence of the melt pool, carbides are also exposed to the direct interaction of the laser beam radiation.

The carbide particles dissolved in the melt pool first enrich the liquid solution with carbon and tungsten and then recrystalize as secondary carbides in the form of dendrites, needle-like or block-like precipitations. So, the optical micrographs of the clad layer LC4 are typical for the clad produced at excessive energy and heat input, characterized by unfavourable microstructure with low share of massive tungsten carbides and high amount of secondary precipitations, Figure 9a,c. On the other hand, the clad layer HC4 produced at the same processing parameters and heat input 240 J/mm but additionally with the localized cooling showed clearly lower tendency for decomposition the massive carbides due to dissipation of the excessive heat by the nitrogen vapours stream under cryogenic conditions. Therefore, the amount and size of precipitations in the regions between massive carbides is lower. The massive carbides show “feather” structure, typical for eutectic W_2_C/WC carbides, Figure 9d.

Beside comparison of microstructure, the optical micrographs revealed a cluster of pores on the cross section of the clad LC4 produced by conventional laser cladding. The pores are placed in the upper part of the clad layer and the average diameter is approx. 20 ÷ 30 µm.

In turn, the optical micrographs of the comparative clad layers produced at minimum heat input of 60 J/mm and different cooling conditions (LC1 and HC1) also show differences in microstructure, Figure 10. First, if compared to the clad layers produced at the maximum heat input, these clad layers have more massive carbides on cross sections and their share in relation to the matrix is significantly greater, Figure 10. Additionally, the carbides are evenly distributed on the cross sections. However, the number and share of massive carbides is clearly greater in the case of the clad layer produced under forced cooling by the localized stream of nitrogen vapours, Figure 10a,b. Observations of the optical micrographs confirmed the tendency for porosity in the case of clad layers produced by conventional laser cladding at free cooling conditions, even at the minimum heat input of 60 J/mm, Figure 10a. The cluster of pores can be observed in the middle part of the clad layer LC1, while the average diameter of pores ranges between 20 to 80 µm. In turn, just two single pores with a diameter approx. 15 µm were observed on cross section of the clad produced under forced cooling conditions, Figure 10d.

In contrast to the clad layer produced at maximum heat input and free cooling, the microstructure of the clad LC1 in the regions between the massive carbides is characterized by a low number of individual precipitations not exceeding 10 µm in length, as can be clearly seen in Figure 10c. There are two explanations for this phenomenon. The first is related with lower amount of decomposed and diluted carbides due to four times lower laser output power (500 W) and thus the heat input. Therefore, the enrichment of the liquid by the W and C elements was also limited and insufficient for precipitation of carbides. The second reason is related with lower volume of the melt pool thus shorter time of material being at liquid state and shorter time for precipitation.

Close observation of the massive carbide’s morphology on the optical micrographs in Figure 10d, showed that all these carbides are covered with a thin layer of block-like particles. Similar observations were reported by Zhang P. et al. [12] for spherical and irregularly shaped carbides in nickel-based matrix. Jones M. and Waag U. provided a comprehensive study on the effect of WC particle type on Ni-based composite coatings. They explained this phenomenon by partial melting of the WC particles during laser cladding. Next the C and W elements combine with Cr, Fe, and Ni to form the complex carbides M_23_C_6_, M_7_C_3_, and M_6_C, which distribute in the matrix to form needle-like or block-like. However, a detailed analysis of this boundary and an attempt to explain this phenomenon is provided in the further part of the discussion.

**Figure 11:**
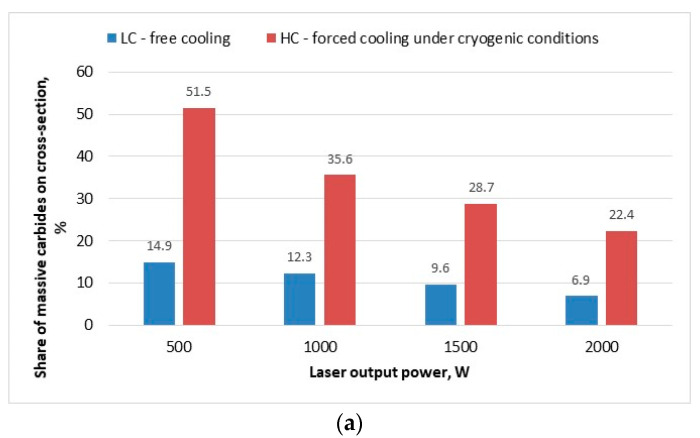

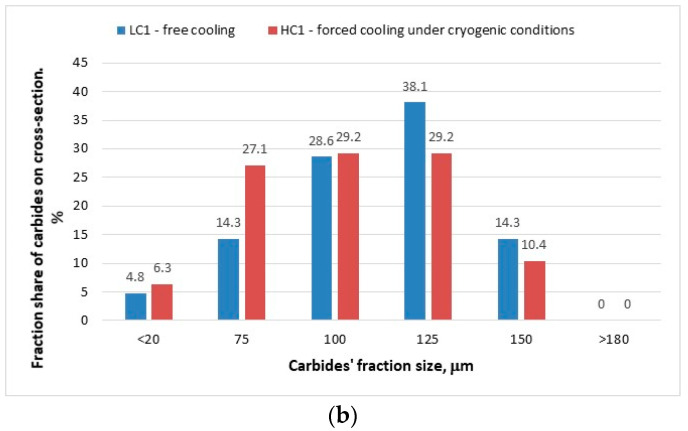
Influence of laser output power and cooling conditions on the share of massive carbides (**a**) and the fraction share of carbides on the cross-section of the clad layers produced at minimum laser out power 500 W at free and forced cooling under cryogenic conditions (**b**).

To quantify the relationship between of laser output power of deposition process (proportional to heat input at constant scanning speed) and the cooling conditions and the morphology of the clad layer, the share of carbides relative to the matrix was determined on the cross sections, and the carbides size distribution for different clad layers was determined. The calculations were made by digitalising the micrographs images and using the NIS-Elements software (Nikon Corporation, Tokyo, Japan) for determining the area share of carbides, and the results are presented in Figure 11.

As can be seen, the calculated percentage share of carbides on cross section of the clad layer produced at maximum heat input of 240 J/mm and free cooling conditions is below 7%, while the share of carbides in the case of clad layer produced at the same heat input but with additional forced cooling is significantly higher, approx. over 22%, Figure 11a.

The relationship indicates generally the lower heat input the higher the share of carbides in the matrix. However, as can be seen, the effect of the forced localized cooling is advantageous, because it provides significantly higher share of carbides for every clad layer produced in the range of investigated parameters. In turn, the carbides size distribution determined for the comparative clad layers produced at minimum heat input of 60 J/mm show that the dominant size for the clad layer LC1 produce under free cooling conditions is approx. 150 ÷ 200 µm. While in the case of the clad layer HC1 produce under forced cooling conditions the carbides distribution is different and the share of carbides sized in the range 50 up to 150 µm is even and proportional, Figure 11b. The relationship clearly indicates that the cooling conditions at the same heat input affect significantly the thermal conditions and the tendency to dissolve carbides and thus reduce their size is lower when forced cooling is applied.

**Figure 12:**
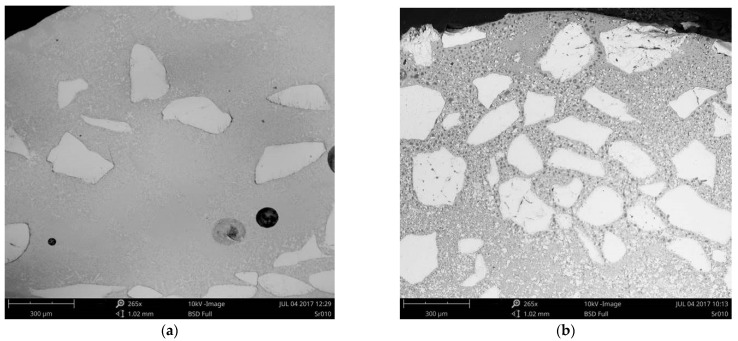
SEM micrographs of the comparative stringer beads LC1 and HC1 produced at the same minimum heat input of 60 J/mm (scanning speed 500 mm/min, laser output power 500 W) but different cooling conditions (Table 3): (**a**) free cooling in ambient air—a view of the entire clad layer and the middle region of the clad respectively; and (**b**) forced localized cooling by nitrogen vapours stream under cryogenic conditions (“hybrid laser deposition”)—a view of the entire clad layer and the middle region of the clad respectively.

**Figure 13:**
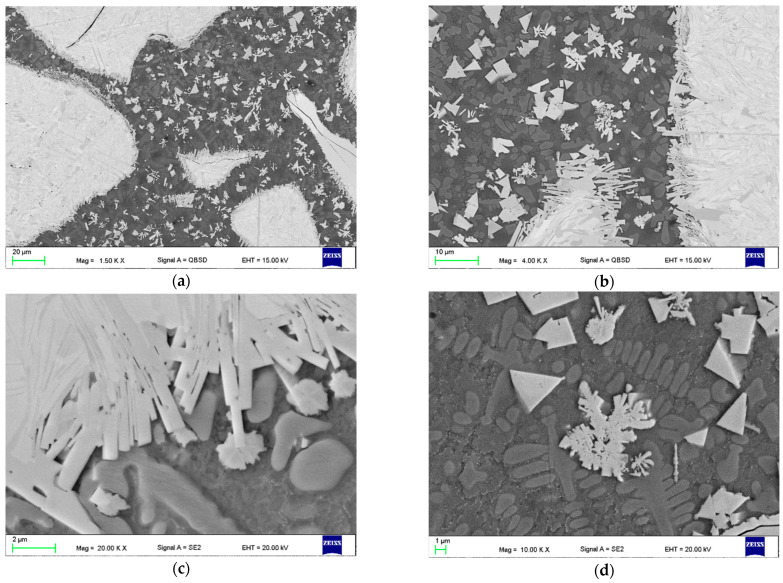
SEM microstructure of the stringer bead HC1 produced at the minimum heat input of 60 J/mm (scanning speed 500 mm/min, laser output power 500 W) and forced localized cooling by nitrogen vapours stream under cryogenic conditions (“hybrid laser deposition”, Table 3): (**a**) general view of massive carbides and matrix; (**b**) a view of carbides boundaries; (**c**) partially recristalized needle-like carbides; (**d**) block-like particles in the dendritic matrix.

**Figure 14:**
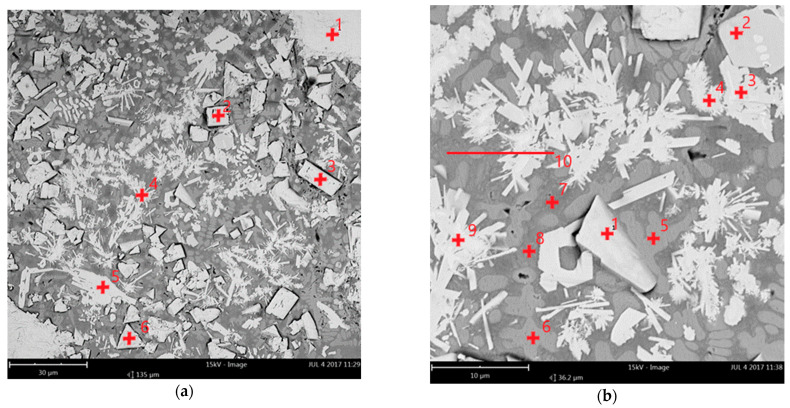
SEM microstructure of the selected representative regions between the massive tungsten carbides on cross section of the stringer bead HC1 produced at the minimum heat input of 60 J/mm (scanning speed 500 mm/min, laser output power 500 W) and forced localized cooling by nitrogen vapours stream under cryogenic conditions (“hybrid laser deposition”, Table 3): (**a**,**b**) a view of the region between massive carbides; results of EDS for individual points and line scan provided in Table 4.

**Figure 15:**
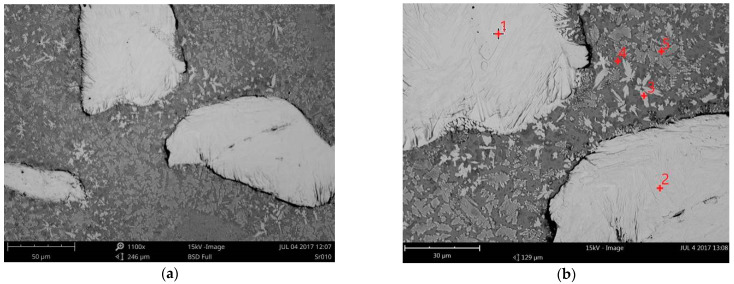

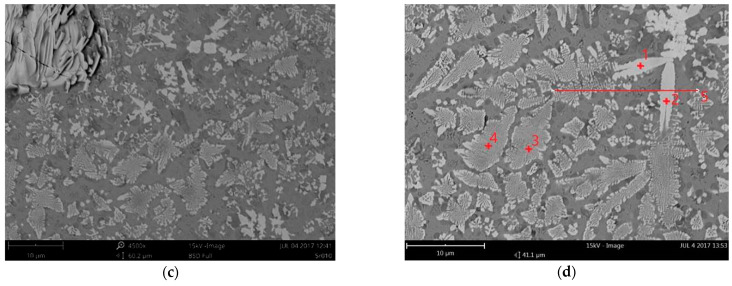
SEM microstructure of the stringer bead LC1 produced at the minimum heat input of 60 J/mm (scanning speed 500 mm/min, laser output power 500 W) and free cooling in ambient air (Table 3): (**a**,**b**) general view of massive carbides and matrix; (**c**) a view of carbides boundaries; (**d**) a view of precipitations in the matrix. Results of EDS for individual points and line scan provided in Table 5.

In turn the size distribution of carbides determined on the surface of the specimen indicates that the proportion of fine carbides is significant, higher than it would appear from the fraction share for the powder. The first reason for this phenomenon is the presence of secondary carbides precipitated in the liquid because of crystallization. However, these are carbides mainly in dendritic form. The share of such dendritic carbides is greater in the range of higher heat inputs (laser power), due to the conditions conducive to the dissolve of carbides in the liquid solution because of diffusion, decomposition due to direct heating with a laser beam, and then precipitation from the liquid solution. However, the presence of a large amount of very small carbide particles is also evident. The explanation for the presence of such many fine carbides is the primary form and morphology of WC-type carbides, which are in fact the eutectic of WC + W_2_C manufactured as a result of melting and crushing. As can be seen from Figure 10d, Figure 12, Figure 13, Figure 14 and Figure 15a,b these massive eutectic carbides are coated by a layer of stoichiometric WC with high thermal stability.

However, in the conditions of laser cladding the particles are exposed to direct action of the laser beam, and thus separation or even decomposition. If the carbide gets directly to the melt pool with a temperature below the carbides melting point, its decomposition will be slight, mainly due to the mutual diffusion of carbide and the liquid. When the carbide is exposed to direct laser beam radiation with high power density, it can directly lead to its partial or complete decomposition. In such a case, the carbide decomposes into fine carbides of needle-like or block-like form, as can be seen in Figure 9b and Figure 14a,b.

The representative and comparative SEM micrographs were presented for the clad layers produced at the minimum heat input and different cooling conditions in Figure 12. The SEM micrograph of the clad layer produced at free cooling in Figure 12a show fine dendritic precipitations mainly near the massive carbides. In turn, on the SEM micrograph of the clad layer produced under forced cooling conditions in Figure 12b the regions between the massive carbides are densely filled mainly with fine block-like particles. The SEM micrographs with higher magnification of the clad layer HC1 produced at forced cooling revealed fine dendritic and needle-like particles, as well as trapezoidal blocks evenly distributed in dendritic matrix, Figure 13. While the massive eutectic W_2_C/WC carbides with feather structure are composed of needle-like and trapezoidal blocks of carbides which are light grey and white, Figure 13a–c. The boundaries of the massive carbides are partially dissolved, as can be seen in Figure 13b,c. It is also characteristic that all needles and trapezoidal blocks on the boundary are light grey. Between the light grey particles at the massive eutectic carbide’s boundary the grey dendrites and dark grey interdendritic regions of matrix can be clearly seen, Figure 13d. It can be also seen that some of the light grey particles, especially long needles (length up to 40 µm), go deep into the massive eutectic carbides, Figure 13b,c. The observed and described morphology of the light grey particles (needle-like and trapezoidal blocks) on the boundary of massive eutectic carbides indicates that the particles were not formed because of recrystallization or precipitation from the liquid phase of the melt pool, but the particles are the integrated part of the massive eutectic carbides, Figure 13.

Another evidence that the light grey particles on the boundaries of massive eutectic carbides were not recrystalized are the very fine crystals growing epitaxially on the tips of some of needles and tops of trapezoidal blocks, as shown in Figure 13c,d. Since the needles’ tips have been exposed to longest exposure to the liquid and higher temperature, therefore, these regions were dissolved and recrystalized. In turn, smaller separate particles (both needles and blocks) almost completely recrystalized into dendrites, as shown in Figure 13b,d. The above observations indicate that just more thermodynamically stable phases (stoichiometric carbides) remained at the boundary of massive eutectic carbides. Since the massive eutectic carbides (eutectic composition lies at approx. 38 at.% C on W-C phase diagram) are originally composed of approx. 80% of W_2_C (β phase) and 20% of stoichiometric WC (δ phase), it can be assumed that the remaining phases on boundary are WC carbides. According to a W-C phase diagram, the melting point of tungsten monocarbide WC is at least 50÷60 degree higher if compared to melting point of W_2_C [32,33]. According to some literature data, the difference is even greater. The melting point for β-W_2_C is often reported at 2730 °C, while for δ-WC at 2870 °C. Moreover, the δ-WC phase is metastable almost up to melting point. However, it should be noted that the conditions of phases transformation during laser deposition are far from the conditions of thermodynamic equilibrium, due to high rates of heating, cooling and high maximum temperature and also high temperature gradients.

In the case of chemical composition analysis with an EDS spectrometer, the quantitative content of elements such as nitrogen and carbon are usually not given due to the high uncertainty and measurement error. However, in the case of the presented results of the study on chemical composition, a satisfactory and sufficient convergence of the results of carbon and nitrogen content was obtained, allowing for the identification and confirmation of the presence of the expected constituents. For this reason, it was decided to include the results of the quantitative measurements of carbon and nitrogen content to ensure greater clarity of the results.

The EDS analysis conducted on the cross-section of the clad layer HC1 produced at the lowest heat input and forced cooling, confirmed that the composition of the massive carbides is typical for eutectic W_2_C/WC, Figure 14, Table 4. In turn, the composition of the trapezoidal block indicates presence of stoichiometric monocrystalline tungsten carbides WC, Figure 14, Table 4. The determined composition of grey dendrites indicates for the matrix of primary γ-Ni, while the dark grey interdendritic regions show composition typical for Ni/Ni_3_B eutectic, and possible share of M_2_C type carbides, Figure 14, Table 4. The EDS analysis conducted for the needle-like constituents indicated composition typical for complex carbide M_7_C_3_ type, Figure 14, Table 4. It should be also noted that the content of iron Fe detected in the clad layer LC1 is relatively low. The Fe was confirmed just in the interdendritic regions, Figure 14, Table 4. It indicates that thanks low dilution of the clad by the substrate material, the composition of the clad is close to the applied powder.

Although the quantitative results of the measurement of nitrogen content should be considered with a great uncertainty, it is evident that the nitrogen was detected in the clad layer produced under cryogenic conditions, mainly in regions of matrix dendrites and interdendritic regions, Figure 14 and Table 4. It is most likely the result of nitrogen vapours stream application directly into the deposition region, and thus the presence of high amount of gaseous nitrogen in this region. In any case, the test results show that there is partial nitrogen absorption under the investigated conditions of laser deposition.

On the other hand, the morphology of the clad layer LC1 produced at minimum heat input but at free cooling conditions observed on the SEM micrographs is completely different that the morphology of the clad layer produced under forced cooling conditions, Figure 15. Beside significantly lower share of massive eutectic carbides, the morphology of precipitations in the regions between the massive eutectic carbides is also different. Closer observation of the massive carbides revealed another difference in the morphology. These carbides observed on the cross-section of the clad layer LC1 produced at free cooling have been dissolved much less in the boundary regions if compared with the carbides of clad layer produced at forced cooling, Figure 14 and Figure 15.

Just little traces of local dissolution and subsequent resolidification can be found on the carbides’ boundary. However, it should be noted that these are just few massive eutectic carbides that have not completely dissolved. The explanation is that the liquid solution has reached the solubility limit of carbon and tungsten, at least locally, which inhibited the further dissolution of remaining massive carbides in the melt pool. The resolidified regions of boundaries show fine light grey dendritic precipitation smaller than 10 µm, Figure 15b,d. In turn, the massive carbides are also composed of needle-like particles and trapezoidal blocks, however, highly integrated and embedded in the matrix-like phase, as can be seen in Figure 15b. It can be supposed that the matrix-like phase is the sub stoichiometric tungsten carbide W_2_C (β phase) which is less stable than the stoichiometric monocarbide WC (δ phase). In Figure 15 there are no traces of fine needle-like particles and trapezoidal blocks typical for the clad layer HC1 produced at forced cooling under cryogenic conditions. This is another evidence that these particles do not nuclei nor solidify in the liquid melt pool under the investigated laser cladding conditions. The particles rather come from the decomposition and dissolution of massive eutectic W_2_C/WC carbides. Since the particles are small and have low heat capacity, thus they heat up intensively and next completely dissolve in the liquid malt pool. However, it must be emphasised that the observations and findings are valid just for the specific conditions of the experiment, determined technological conditions such as the laser beam characteristic (energy density, intensity profile, wavelength, etc.), processing parameters, the way of powder delivery, thermal conditions.

The EDS analysis conducted on the cross-section of the clad layer LC1 produced at the lowest heat input and free cooling, beside the massive eutectic W_2_C/WC, confirmed presence of M_2_C type carbides as dendritic precipitations, Figure 15 and Table 5. In turn, the densely packed light grey precipitations between the massive carbides show composition typical for M_3_C carbides in eutectic, Figure 15, Table 5.

The composition of dark grey matrix indicates presence of Ni/Ni_3_B eutectic, possibly with some amount of M_2_C type carbides, Figure 15, Table 5. In general, the dilution was higher from a dozen to several dozen percent in the case of clad layers produced at free cooling conditions, Figure 8. Therefore, the elements from the non-alloy steel substrate, mainly the Fe, passed into the clad. As a result of higher dilution, the composition of the clad is different than the original composition of the applied powder, Table 2.

Although the quantitative results of the measurement of nitrogen content should be considered with a great uncertainty, it is evident that in this case the nitrogen content is significantly lower, on the limit of the measurement error of EDS spectrometer, Table 5. This is due to application of shielding of argon during laser deposition tests, and thus protection against absorption of gases from the ambient. The X-ray diffraction pattern of the experimental powder is presented in Figure 3. It indicates the presence of tungsten carbides δ-WC and β-W_2_C, as well as the γ-Ni. Additionally, clear picks for Ni_3_B and complex carbides M_7_C_3_ type were identified, Figure 3.

**Figure 16:**
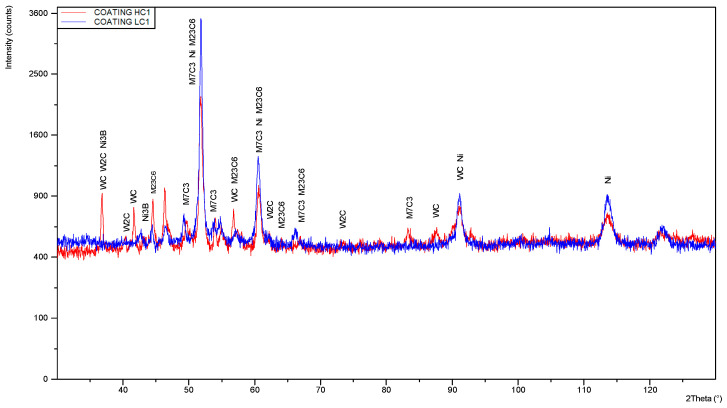
XRD patterns of the comparative coatings produced at the same minimum heat input of 60 J/mm (scanning speed 500 mm/min, laser output power 500 W) but different cooling conditions: LC1 at free cooling, and HC1 with forced localised cooling under cryogenic conditions, Table 3.

In turn, the X-ray diffraction patterns for two comparative samples are presented in Figure 16. The comparative samples were prepared by multi-bead cladding of the disc’s substrate intended for the tribological tests. The discs were produced by cladding the substrate with an overlap approx. 20 ÷ 25%, to produce the coating on the entire disc surface. The comparative coatings were produced at the minimum heat input (minimum laser output power) at free cooling and at forced cooling under cryogenic conditions (parameters for the single beads LC1 and HC1, Table 3). Surfaces of the discs were grinded with roughness Ra 0.8 ÷ 1.1 μm.

The initial and general comparison of the two X-ray diffraction patterns show a clear difference in the phase composition. The sample coated at forced cooling under cryogenic conditions (parameters for HC1) shows simply more peaks compared to the sample coated at free cooling (parameters for LC1), Figure 16. However, peaks for the LC1 are more intensive. It is evident that the peaks for γ-Ni are dominant in the case of the coating produced at free cooling (LC1), Figure 16. There are also clear peaks for complex carbides M_23_C_6_ and M_7_C_3_ type. Moreover, slight peaks for δ-WC and Ni_3_B were identified. The obtained results are consistent with the previous observations of SEM micrographs and EDS analysis. The phase composition of the clad layer produced under free cooling LC1 is also affected by the higher dilution by the substrate material, mainly iron Fe, as shown the chemical composition analysis. In turn, the X-ray diffraction patterns indicate that the share of eutectic W_2_C/WC is significantly higher in the coating produced under cryogenic conditions. The dominant γ-Ni phase in the coating produced at free cooling is a result of different thermal conditions of the deposition process, higher impact of the heat, and thus higher degree of carbides decomposition and dissolution, as well as higher dilution by the Fe from the substrate of non-alloy steel. Therefore, the carbides precipitated from the liquid solution are more complex, as shown in Figure 16.

It should be noted that the detection level of the applied XRD method is approx. 3% for the individual phase, therefore, it does not allow for precise identification of the fine precipitations at a low share. However, due to the conjunction with chemical composition analysis in the micro regions and individual constituents, precise detection of the phase composition was provided.

### 3.3. Hardness

**Figure 17:**
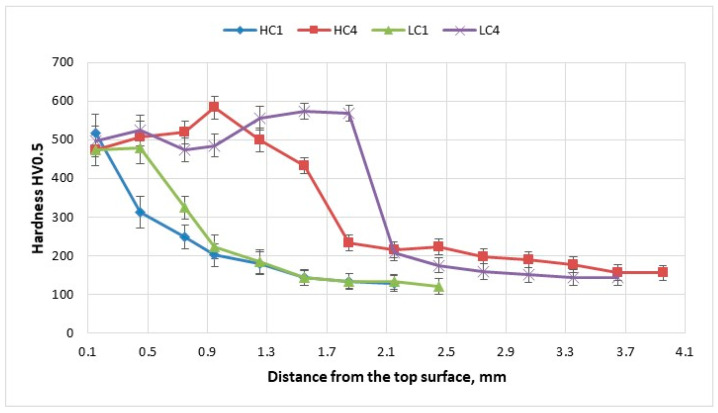
Hardness distribution on cross-sections of the representative clad layers produced by laser deposition of experimental WC-Ni composite powder (Table 3) at free cooling conditions (LC1, LC4—conventional laser cladding); and at forced localized cooling by nitrogen vapours stream under cryogenic conditions (HC1, HC4—hybrid laser deposition process).

The profiles of hardness were determined on cross-section of the clad layers produced at minimum and maximum heat input at free cooling and under cryogenic conditions. For the Vickers tests of the composite clad layers the applied load was set at 5N. The large load was chosen to avoid large scatter of results (low values for matrix and high for carbides) providing mean hardness value for the subsequent regions. However, the profiles of Vickers HV0.5 hardness presented in Figure 17 showed clear difference for the hardness distribution for individual clad layers. The difference is related mainly with the width of the zone with a high hardness. The clad layers produced at free colling show higher width of the zone with the high hardness ranged approx. from 500HV0.5 to 600 HV0.5. It is related directly with the shape of the individual clads, specifically with the height and penetration depth. The clad layers produced at free cooling conditions have higher penetration depth compared to the clads produced under cryogenic conditions. Therefore, the total depth (width on the graph) of the clad layers produced at free cooling conditions is higher.

On the other hand, despite the difference in the chemical and phase composition of the clad layers, the maximum values of hardness and the scattering of results are very similar. It should be noted that the presented results of hardness measurement for each point on the graphs are a mean value taken from three different sections for every tested clad layer. The values of standard deviation indicate small dispersion of results. This is due to relatively large size of Vickers’s indenter imprint at the load of 5N. The clad layers HC1 produced al lowest heat input and under cryogenic conditions showed the highest value of hardness 520HV0.5 directly under the top surface (face) of the clad. In turn, the highest measured hardness approx. 600HV0.5 was detected for the clad layer produced at maximum het input and cryogenic conditions. For every tested clad layer, starting from a certain depth the hardness decreases gradually till the value of the base metal of non-alloy steel (S235JR) approx. 120 ÷ 15HV0.5. This finding contrasts with the phenomenon described in the previous manuscript on “Laser Deposition of Fe-based metallic powder under cryogenic conditions” [1]. However, different technique of cryogenic cooling was applied in the previous study, consisted of submerging the entire sample in the liquid nitrogen bath. Therefore, the undercooling of the substrate was higher in the previously tested “liquid nitrogen bath” technic.

### 3.4. Tribological Test

**Figure 18:**
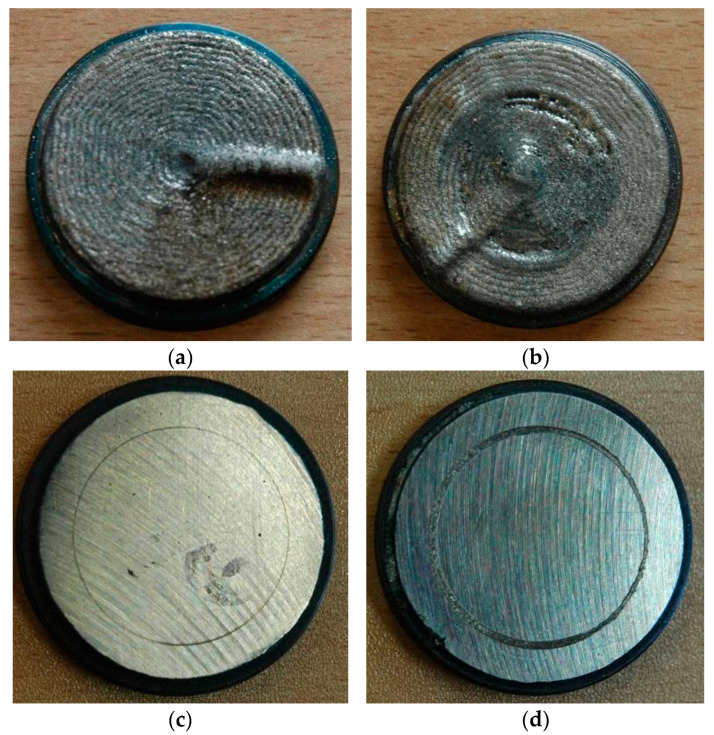
A view of the discs prepared for ball-on-disc wear tests with coating produced by laser deposition; (**a**) coating produced with parameters and technological conditions for the clad HC1 (cryogenic hybrid method), (**b**) coating produced with parameters and technological conditions for the clad LC1 (conventional laser cladding), and after the tribological test; (**c**) sample with coating HC1 type, and (**d**) sample with coating LC1 type.

It must be noted that the task of the study is not a comprehensive analysis of the mechanisms of wear and tribological characteristics. Therefore, just basic results of tribological ball-on-disc tests were presented for the final comparison of the characteristics and wear resistance of the comparative coatings produced under different technological conditions and different thermal conditions. The values presented at the graphs are mean values taken from at least three individual measurements. The volume loss of the coatings was calculated by determining the wear track profile in four places around the circumference of the wear track. Comparison of the wear tracks for representative samples produced at the lowest heat input but different cooling conditions (coating produced at parameters for HC1 and LC1) is shown in Figure 18.

**Figure 19:**
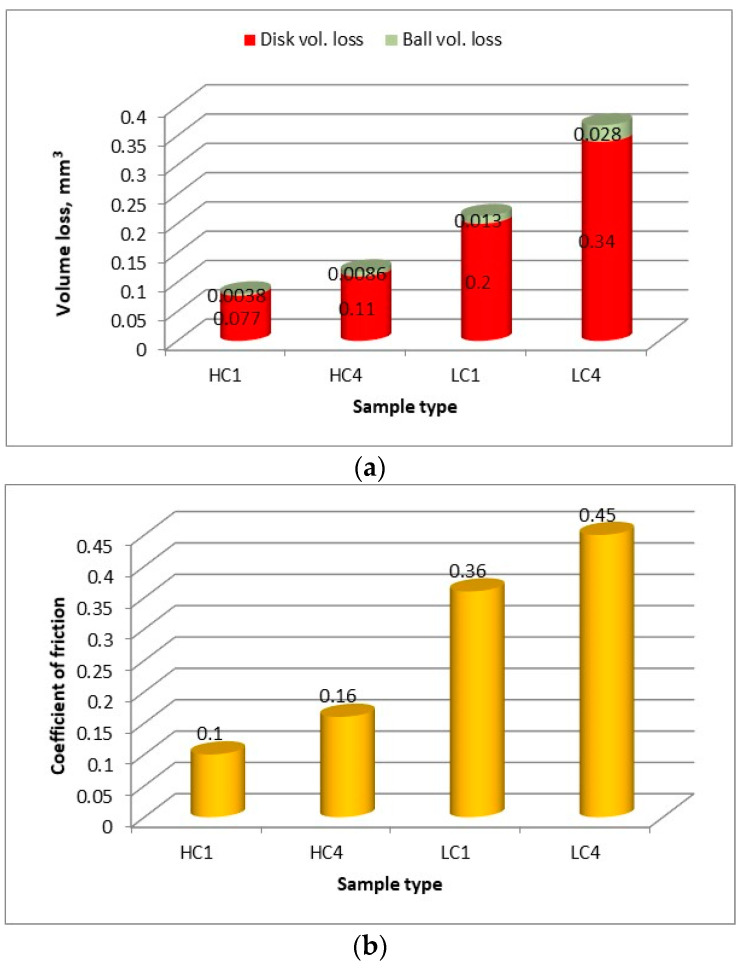
Volume loss of the counter body steel ball and the disc specimens of commercially pure titanium, titanium alloy and the nitrided surface layers and after sliding for a distance of 188.4 m at normal load of 30 N (**a**), and comparison of the coefficient of friction of the disc specimens sliding with a steel ball during ball-on-disc wear tests (**b**).

The wear track of the sample HC1 produced under cryogenic conditions and the lowest heat input has the smallest width, as shown in Figure 18c. As expected, the determined volume loss was also the lowest for the coating HC1, Figure 19a. Since the lowest volume loss, the better wear resistance, it means that the composite coating produced at the lowest heat input and cryogenic conditions have the highest wear resistance under dry sliding conditions of the experiment. As can be seen in Figure 19a, even the coating produced at the maximum heat input but at localized forced cooling under cryogenic conditions showed lower volume loos and thus higher wear resistance than the coatings produced at free cooling and the lowest heat input. An explanation for this observed phenomenon can be found in Figure 11a, showing the share of massive carbides in the matrix. The share of massive carbides (eutectic W_2_C/WC) in the matrix of every clad layer produced under cryogenic conditions is higher than for the clad layers produced at free cooling. Even in the case of the clad layer produced at the maximum heat input HC4 (laser output power 2000 W) but with simultaneous localized cooling the share of massive carbides is 22.4%, while in the case of the clad layer produced at the lowest heat input LC1 (500 W) but free cooling the share of carbides is less than 15%. Thus, the results show that under the test conditions, the wear resistance of the composite coatings is proportional to the massive carbides (eutectic W_2_C/WC) content in the matrix. On the other hand, the volume loos of the counter face material of steel ball is directly proportional to the volume loos of the discs samples. This phenomenon may be explained by the values of the coefficient of friction determined during individual tests. The values of coefficient of friction presented at the graph in Figure 19b are the mean values, however, a clear relationship between the coefficient of friction and wear intensity can be observed. In general, the higher coefficient of friction the higher wear intensity, both the sample and the steel ball, Figure 19. The obtained results indicate that the coefficient of friction also depends on the phase composition of the coatings, mainly on the share of massive carbides in the matrix, as well as composition of the matrix.

## 4. Conclusions

The novel technique of laser deposition of composite WC-Ni powder with forced localized cooling of the deposit by nitrogen vapours stream under cryogenic conditions was successfully demonstrated and its potential for shaping the geometry and the microstructure was presented. The results of the study confirmed the advantageous effect of the forced cooling in terms of chemical and phase composition, microstructure, and wear resistance under dry sliding conditions. The use of the forced cooling technique significantly reduces the tendency to decomposition and to dissolve carbides in the melt pool during laser deposition of composite WC/Ni powder. The share of carbides on cross section of the clad layer produced at minimum heat input (laser output power 500 W and scanning speed 500 mm/min) at conventional conditions of free cooling was less than 15%, while thanks to the application of forced localized cooling of the deposit the share of carbides was maintained over 51%. Moreover, the degree of dilution was significantly reduced thanks to the forced localized cooling. The chemical composition of clad layer produced at minimum heat input and with forced cooling under cryogenic conditions was very similar to that of experimental composite WC-Ni powder, except for the increased nitrogen content in the dendritic matrix and interdendritic regions. The increased content of nitrogen detected in the matrix of the clad layer is resulted by presence of high amount of gaseous nitrogen in deposition region if the nitrogen vapours stream is applied. Therefore, during the laser deposition of composite powder (laser cladding) the alloying process takes place additionally. The material of the clad layer (the deposit) is simultaneously enriched by nitrogen from the gaseous atmosphere, so the demonstrated process of laser deposition of composite powder with simultaneous localized cooling by the nitrogen vapor stream can be considered as hybrid process, combining the conventional laser cladding (LC) and also laser gas nitriding (LGN).

## Figures and Tables

**Table 1:** Chemical composition of non-alloy structural steel S235JR (EN 10025-2) based on the supplier’s (Thyssenkrupp, Dąbrowa Górnicza, Poland)) certificate (wt %).

C	Mn	Si	P	S	N	Cu	Al	Fe
0.05–0.14	0.2–0.8	0.1	0.025	0.015	0.01	0.2	0.015–0.08	Bal.

**Table 2:** Nominal composition of experimental powder as a mixture of tungsten carbides with nickel-based metal matrix (wt %), Figure 2 and Figure 3.

WC	Fe	Si	B	C	Ni
60	2.0	3.0	3.0	0.02	Bal.

**Table 3:** Parameters of laser deposition of experimental composite WC-Ni powder at free cooling conditions (conventional laser cladding) and forced localized cooling by nitrogen vapours stream under cryogenic conditions (hybrid laser deposition process), Figure 6.

No.	Surface Layer Indication Free Cooling/Hybrid	Scanning Speed (mm/min)	Laser Power (W)	Energy Input * (J/mm)	Remarks Free Cooling/Hybrid
1	LC1/HC1	500	500	60	SP/HQ
2	LC2/HC2	500	1000	120	SP, HQ/HQ
3	LC3/HC3	500	1500	180	SP, HQ/HQ
4	LC4/HC4	500	2000	240	SP/SP, HQ

Remarks: UB—uneven bead, SP—single pore, IF—incomplete fusion, V—voids, HQ—high quality, LF—lack of fusion. * energy input is calculated by simply dividing the laser power by scanning speed, while the heat input should also include the heat transfer efficiency. Other process parameters; powder feeding rate: 8.5 g/min, diameter of the nozzle tip for nitrogen vapours stream: 5.0 mm, average consumption of liquid nitrogen: 80 ÷ 100 g/min.

**Table 4:** Summary of EDS analysis on the cross section of the stringer bead HC1 produced at the minimum heat input of 60 J/mm and forced cryogenic cooling conditions (hybrid laser deposition process), Figure 14.

Point	W	Ni	Si	Fe	C	N	B	Possible Phase
	Atomic/Weight Concentration							
a_1	60.4/95.5	0.8/0.4	-	-	38.8/4.1	-	-	W_2_C/WC
a_2	59.2/94.4	2.7/1.4	-	-	38.1/4.2	-	-	W_2_C/WC
a_3	60.9/95.1	2.0/1.0	-	-	37.1/3.9	-	-	W_2_C/WC
a_4	56.9/90.5	7.7/3.9	2.1/1.1	-	26.4/2.5	3.1/0.5	3.8/1.5	M_7_C_3_
a_5	61.4/92.9	8.0/4.0	-	-	30.6/3.1	-	-	M_2_C
a_6	56.4/94.3	1.8/0.9	-	-	42.0/4.7	-	-	WC
b_1	58.1/94.6	2.0/1.0	-	-	39.9/4.4	-	-	WC
b_2	60.9/91.9	10.0/5.0	-	-	29.1/3.1	-	-	M_2_C
b_3	55.7/90.2	11.5/6.2	-	-	32.8/3.6	-	-	M_2_C
b_4	59.7/92.7	7.1/3.7	-	-	33.2/3.7	-	-	M_2_C
b_5	21.1/54.0	49.8/40.7	-	-	21.9/3.7	7.2/1.6	-	Primary γ-Ni
b_6	21.0/54.5	48.3/39.9	-	-	23.2/3.9	7.5/1.7	-	Primary γ-Ni
b_7	6.0/25.7	37.8/52.3	4.5/2.9	0.9/1.1	17.4/5.0	27.7/10.6	3.0/2.36	Ni/Ni_3_B eutectic, M_2_C
b_8	9.9/33.3	54.4/58.3	-	-	26.8/5.9	-	8.8/2.6	Ni/Ni3B eutectic, M_2_C
b_9	64.4/93.6	7.7/3.7	-	-	27.9/2.7	-	-	M_7_C_3_
b_10	32.1/69.7	36.8/25.4	-	-	20.1/2.8	11.0/2.1	-	Line scan

**Table 5:** Summary of EDS analysis on the cross section of the stringer bead LC1 produced at the minimum heat input of 60 J/mm and free cooling conditions (conventional laser cladding), Figure 15.

Point	W	Ni	Si	Fe	C	N	B	Possible Phase
			Atomic/Weight Concentration					
b_1	54.4/94.8	-	-	-	45.6/5.2	-	-	W_2_C/WC
b_2	54.7/94.5	-	-	-	45.3/5.5	-	-	W_2_C/WC
b_3	39.0/82.1	6.6/4.6	9.1/1.7	10.3/6.7	35.0/4.9	-	-	M_2_C
b_4	8.2/31.2	20.5/24.8	5.9/1.8	27.6/31.9	28.9/7.2	-	8.8/3.1	Ni/Ni_3_B eutectic, M_2_C
b_5	22.6/60.7	16.3/14.0	-	21.7/17.7	26.7/4.7	-	12.5/2.9	M_3_C in eutectic
d_1	35.0/80.0	7.6/5.5	11.5/2.3	9.7/6.8	36.2/5.4	-	-	M_2_C
d_2	34.8/80.5	6.7/5.0	16.6/3.3	8.8/6.2	33.0/5.0	-	-	M_2_C
d_3	19.4/57.3	17.6/16.6	-	18.5/16.6	30.0/5.8	-	14.6/3.7	M_3_C in eutectic
d_4	15.8/51.4	17.6/18.3	-	19.0/18.9	28.5/6.0	-	19.1/5.4	M_3_C in eutectic
d_5	18.0/53.5	18.9/17.9	9.6/2.4	21.2/19.2	22.5/4.3	-	10.1/2.7	line scan

## Data Availability

The data presented in this study are available on request from the corresponding author. The data are not publicly available due to know how protection.
